# Mitochondrial chaperonin DNAJC15 promotes vulnerability to ferroptosis of chemoresistant ovarian cancer cells

**DOI:** 10.1098/rsob.240151

**Published:** 2025-01-15

**Authors:** Stefano Miglietta, Manuela Sollazzo, Iacopo Gherardi, Sara Milioni, Beatrice Cavina, Lorena Marchio, Monica De Luise, Camelia Alexandra Coada, Marco Fiorillo, Anna Myriam Perrone, Ivana Kurelac, Giuseppe Gasparre, Luisa Iommarini, Anna Maria Ghelli, Anna Maria Porcelli

**Affiliations:** ^1^ Department of Pharmacy and Biotechnology (FABIT), University of Bologna, Bologna, Italy; ^2^ Centre for Applied Biomedical Research (CRBA), University of Bologna, Bologna, Italy; ^3^ Department of Medical and Surgical Sciences (DIMEC), University of Bologna, Bologna, Italy; ^4^ Department of Pharmacy, Health and Nutritional Sciences, University of Calabria, Arcavacata di Rende, Italy; ^5^ IRCCS Azienda Ospedaliero-Universitaria di Bologna, Bologna, Italy; ^6^ Centro Studi e Ricerca Sulle Neoplasie Ginecologiche (CSR), University of Bologna, Bologna, Italy; ^7^ IRCCS Istituto delle Scienze Neurologiche di Bologna, Programma di Neurogenetica, Bologna, Italy; ^8^ Department of Pharmacy and Biotechnology (FABIT) and Interdepartmental Centre for Industrial Research ‘Scienze Della Vita e Tecnologie per La Salute’, University of Bologna, Bologna, Italy

**Keywords:** DNAJC15, ovarian cancer, cisplatin resistance, ferroptosis, mitochondria

## Abstract

DNAJC15 is a mitochondrial TIMM23-related co-chaperonin known for its role in regulating oxidative phosphorylation efficiency, oxidative stress response and lipid metabolism. Recently, it has been proposed that the loss of DNAJC15 correlates with cisplatin (CDDP)-resistance onset in ovarian cancer (OC), suggesting this protein as a potential prognostic factor during OC progression. However, the molecular mechanisms through which DNAJC15 contributes to CDDP response remains poorly investigated. Here, we show that high levels of DNAJC15 are associated with accumulation of lipid droplets, decreased tumorigenic features and increased sensitivity to CDDP in OC cells. When overexpressed, DNAJC15 induced a phenotype displaying increased lipid peroxidation and subsequent ferroptosis induction. To prove a role for DNAJC15-induced ferroptosis in promoting sensitivity to CDDP, we reduced lipid peroxidation upon Ferrostatin 1 treatment, which decreased cells’ vulnerability to ferroptosis ultimately recovering their CDDP-resistant phenotype. In conclusion, our study uncovers the role of DNAJC15 in modulating ferroptosis activation and in the onset of CDDP resistance in OC cells.

**Figure:**
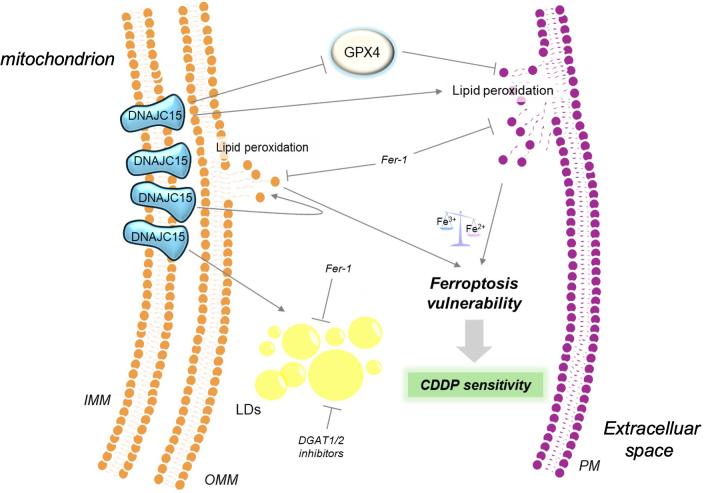

## 1. Introduction

DnaJ heat shock protein family (Hsp40) member C15 (DNAJC15), also known as methylation-controlled J protein (MCJ), is a co-chaperon belonging to the DNAJC subfamily, encoded by the *DNAJC15* gene. This protein is localized in the inner mitochondrial membrane where it interacts with the TIMM23 translocase complex, enhancing the ATPase activity of mitochondrial heat shock protein 70 [1], and favouring the transport of proteins lacking a mitochondrial targeting sequence, thus contributing to the biogenesis of these organelles [2]. Several functions have been inferred for DNAJC15 in the regulation of mitochondrial physiological processes. For instance, DNAJC15 has been suggested to work as a negative endogenous regulator of the respiratory chain since its loss leads to an increase of complex I activity, mitochondrial membrane potential and ATP production [3]. Moreover, low DNAJC15 levels promote fatty acid β-oxidation, thereby decreasing pathological lipid accumulation in the liver [4]. Depending on the cellular context, DNAJC15 has also been described as either a promoter or an inhibitor of oxidative stress [5–7]. Lastly, its involvement in the opening of the mitochondrial permeability transition pore (MPTP) complex and, in turn, in the induction of apoptosis, has led to envisioning this chaperone as a player in the regulation of cell proliferation and death [2,5].

In recent years, several mitochondrial chaperonins have been implicated in the onset of cancer therapy resistance [8–10]. In this frame, suppression of *DNAJC15* expression due to methylation of its promoter CpG islands as well as of the 5′ of the coding sequence has been reported in breast, ovarian, neuroblastoma and brain cancers, and associated with a poor prognosis, also likely thanks to its contribution in promoting chemoresistance [11–16]. One of the mechanisms linking the response to therapy to the loss of DNAJC15 expression is through the fuelling of multi-drug resistance protein activity, with subsequent enhanced drug efflux, as shown in breast cancer [17]. Concomitantly, high DNAJC15 expression levels have been associated with increased responsiveness to paclitaxel, topotecan and cisplatin (CDDP) in ovarian cancer (OC) [5,12,18]. The latter, particularly, presents cogent and unmet clinical needs, due to its high mortality rate, onset of chemoresistance, and low overall and disease-free survival (https://seer.cancer.gov/statfacts/html/ovary.html) [19,20]. Adding to OC complex care is the paucity of therapeutic choices besides debulking surgery, platinum-derived compounds and taxanes are the first-line chemotherapy, to which too often patients stop responding towards poor clinical outcomes [21]. It is well known that such conventional therapeutic strategies in oncology may also trigger alternative cell death modalities such as ferroptosis. Indeed, not only does the latter suppress tumour growth, but also holds potential for surmounting resistance to existing cancer therapies [22]. Ferroptosis is induced by an accumulation of lipid peroxides in cellular membranes mediated by an iron oxidation status unbalance [22]. Notably, mitochondria are the largest stores of this metal in cells and during ferroptosis they undergo alterations in terms of morphology, dynamics, energy metabolism and oxidative stress, pointing out the involvement of mitochondria in this type of cell death [23]. In this study, we exploited CDDP chemoresistant and sensitive OC cells, which concordantly presented with low and high DNAJC15 expression, respectively. We demonstrated that high DNAJC15 levels influence tumorigenic properties in two-dimensional (2D) and three-dimensional (3D) models and stimulate lipid peroxidation and ferroptosis activation. This iron-mediated unprogrammed cell death increases OC cells’ vulnerability to CDDP toxicity, unveiling that DNAJC15 may regulate an alternative anti-proliferative process besides apoptosis to prevent cancer cell growth. This finding may contribute to develop new therapeutic opportunities, a cogent challenge in OC care. Indeed, it is the most lethal and silent gynaecological malignancy since, despite the early positive response to standard treatment based on the combination of platinum and taxane compounds, over time, about 80% of patients develop relapse, resistance with a poor prognosis [19,21].

## 2. Material and methods

### 2.1. Cell model generation, maintenance and treatments

Human OC cell lines A2780 (RRID: CVCL_0134), A2780cis (RRID: CVCL_1942) and SKOV3 (RRID: CVCL_0532) were purchased from ATCC (Manassas, VA, USA), while OC314 (RRID: CVCL_1616) was kindly gifted by Prof. Ada Funaro (University of Turin). A2780cis is a CDDP-resistant cell line, developed by chronic exposure of the parental CDDP-sensitive A2780 cell line. CDDP-resistant SKOV3 cell line (referred to as SKOV3cis) was generated by chronic CDDP (Cayman Chemical, 13119) treatments (2–8 µM) of the parental SKOV3 cell line. Stable DNAJC15 overexpression in A2780cis and SKOV3cis cells (referred to as DNAJC15-OE) was performed through lentivirus-mediated infection, by using lentiviral ORF particles (*DNAJC15* Myc-DDK tagged) (Origene, RC210567L3V) or lentiviral ORF control particles (mock) (Origene, PS100092V), following manufacturer instructions. Stable DNAJC15 downregulation in OC314 cell line was performed through lentivirus-mediated infection (Origene, TL313420), by using viral particles containing the shRNA plasmids for *DNAJC15* silencing (referred to as DNAJC15-KD) or empty vector (scramble). For each shRNA, lentiviral particles were generated following manufacturer instructions, using 5 µg of shRNAs and 6 µg of packaging plasmids (Origene, TR30037). All the infected cell lines subsequently underwent antibiotic selection in 0.75 μg ml^−1^ puromycin followed by clonal selection, which allowed us to obtain three clones for each cell line that were subsequently pooled.

All cell lines were cultured in RPMI 1640 medium (Gibco™ Thermo Fisher Scientific, 21875034) supplemented with 10% FBS (Gibco™ Thermo Fisher Scientific, 10270106), 1% penicillin/streptomycin (Gibco™ Thermo Fisher Scientific, 15070063) and maintained at 37°C in a humidified atmosphere with 5% CO_2_. Growth medium was supplemented with 0.125 μg ml^−1^ puromycin to maintain the expression of exogenous DNAJC15 as well as DNAJC15-shRNA. Cell lines were regularly tested for mycoplasma contamination.

CDDP acute treatments were performed for cell viability assay to determine CDDP half-maximal inhibitory concentrations (IC_50_), with a range of concentrations spanning from 0.5 to 20 μM. The resistance of CDDP-resistant OC cells was periodically checked and maintained by administering every eight passages CDDP at a concentration corresponding to the respective IC_50_ for 72 h, followed by a recovery in fresh culture medium. Combined treatments with DGAT1 inhibitor A922500 (Sigma-Aldrich, A1737) and DGAT2 inhibitor PF-06424439 (Sigma-Aldrich, PZ0233) were performed at 10 μM final concentration, for 24 h for lipid droplet staining or until 48 h and 72 h for western blot and cell viability assays, respectively. Ferrostatin-1 (Sigma-Aldrich, SML0583) treatments were performed administering 15 μM of the drug for 24 h for western blot analysis or for 72 h for cell viability assay and spheroid experiments.

### 2.2. Cell viability assay

Cell viability was evaluated by the sulforhodamine B (SRB) method [24]. Briefly, cells were seeded in 24-well (15 000 cells per well) or 96-well (5000 cells per well) plates. Then, 24 h after seeding, cells were treated as specified in each panel and the cell growth was monitored for 72 h. For each analysed time point (24, 48 and 72 h), cells were fixed adding 10% trichloroacetic acid (TCA) and incubating them at 4°C for 1 h. Thereafter, cells were washed five times with distilled water to remove TCA and dried at room temperature (RT). Next, SRB solution (0.4% in 1% acetic acid) was added to each well and incubated for 30 min at RT. After incubation, unbound SRB dye was removed by washing four times with 1% acetic acid. Finally, the dye was solubilized by adding 10 mM Tris-base (pH 10.5). SRB absorbance was measured at 560 nm using a VICTOR^3^ 1420 Multilabel Counter plate reader (Perkin-Elmer). CDDP IC_50_ was determined using GraphPad Prism and calculated as concentration that impacts on 50% cell viability in comparison with untreated sample.

### 2.3. Caspase-3/7 activation assay

Apoptosis was evaluated using Incucyte^®^ S3 Live-Cell Analysis System (Sartorius). Cells (5000 cells per well) were seeded in a 96-well plate. After 24 h, cells were incubated with 5 μM Incucyte^®^ Caspase-3/7 Dye for Apoptosis (Sartorius, 4440). Then, the plate was placed in Incucyte^®^ for 72 h and images (4 per well) were captured at 10× magnification with 4 h intervals, both in phase contrast and fluorescent green channel. For each cell line, staurosporine (100 nM, Sigma-Aldrich, S4400) treatment was performed as a positive control. For each cell line, time point and experimental condition, the masks for fluorescent object identification and total cell number count were set by the Incucyte^®^ integrated analysis software. Total cells were counted using adherent cell-by-cell analysis software. Data were expressed as percentage of apoptotic cells ((number of green objects/total cell number) × 100) subtracted fromto the percentage of apoptotic cells at 0 h.

### 2.4. Clonogenic assay

The ability to form colonies was evaluated by seeding 300 cells per well for each cell line in 6-well plates. Ten days after seeding, colonies were stained using SRB as described in §2.2 and images were acquired using a Gel Logic 1500 Imaging System (Kodak). SRB absorbance was measured at 560 nm using VICTOR^3^ 1420 Multilabel Counter plate reader (Perkin-Elmer).

### 2.5. RNA extraction and quantitative real-time polymerase chain reaction

Cells were seeded in 6-well plates (150 000 cells per well) and harvested after 48 h after trypsinization. RNA was extracted from cell pellets using RNeasy^®^ Mini Kit (Qiagen, 74106) following manufacturer’s instructions, eluted in 30 µl of RNase-free water and quantified using a NanoDrop™ 2000 (Thermo Scientific). Then, 300 ng of RNA was retrotranscribed into cDNA using a High-Capacity cDNA Reverse Transcription Kit (Applied Biosystems, 4368814) with random hexamers following manufacturer’s instructions. Quantitative real-time PCR (qRT-PCR) was performed using SYBR Green. The primer sequences for the SYBR Green assay were designed using Primer3 software (https://primer3.ut.ee). The primers for *DNAJC15* span the second and third exon of the coding sequence allowing the recognition of both the endogenous and the exogenous *DNAJC15*. The formation of homo- and hetero-dimers was evaluated using the IDT OligoAnalyzer tool (https://eu.idtdna.com/analyzer/Applications/OligoAnalyzer), and cDNA secondary structures were estimated using the Mfold web server (http://www.unafold.org). SYBR Green assays were performed using GoTaq qPCR Master Mix (Promega, A6002) in a 7500 Fast Real-Time PCR System (Applied Biosystem), following manufacturers’ instructions. The amplification reactions were carried out using the following conditions: 95°C for 5 min (holding stage); 40 cycles of 95°C 15 s and 60°C 45 s (cycling stage) and melting curve of the resulting amplicons was analysed to rule out non-specific amplifications. To calculate the relative gene expression for each biological replicate, the 2^−ΔΔCt^ method was used, with the following formula: 2^−[ΔCt (sample) − ΔCt (control)]^, where ΔCt = [Ct (gene of interest) − Ct (reference gene)]. For each experimental point, data are represented as fold change (FC) relative to the average ΔCt value of control samples. *HPRT* was used as a reference gene. The statistical significance between conditions was assessed using a *t*‐test, calculated on the FC values. *DNAJC15* primers pair: Fw: 5′-TTTCGGATCTGGAAACCTCTAG-3′; Rv: 5′-TCTCGCCTACTCATTTTCTGTT-3′. *HPRT* primers pair: Fw: 5′-CATTGTAGCCCTCTGTGTGC-3′; Rv: 5′-CCACCAATTACTTTTATGTCCCC-3′.

### 2.6. Mitochondrial enriched-fraction preparation

Mitochondrial fractions were isolated from 20 to 40 × 10^6^ cells, suspended in sucrose–mannitol buffer (200 mM mannitol, 70 mM sucrose, 1 mM EGTA and 10 mM Tris–HCl at pH 7.6) and homogenized using a glass/Teflon Potter-Elvehjem homogenizer. Differential centrifugation (600 × *g* for 10 min at 4°C followed by 10 000 × *g* for 20 min at 4°C) was performed to separate crude mitochondria from other subcellular fractions. The resulting pellets were stored at −80°C and used for SDS-PAGE and western blot analysis.

### 2.7. SDS-PAGE and western blot

Protein extraction was obtained by resuspending mitochondrial fraction and total cellular pellets in RIPA buffer (50 mM Tris–HCl (pH 7.4), 150 mM NaCl, 1 mM EDTA, 1% Triton-X, 0.1% SDS) supplemented with protease inhibitors (Roche, 11697498001), incubating for 15 min at 4°C and then by freezing and thawing samples twice. Extract protein content was quantified using the Bradford method. Samples were prepared adding loading dye 5× (300 mM Tris–HCl (pH 6.8), 12.5% β-mercaptoethanol, 10% SDS, 0.125% bromophenol blue, 2.5% glycerol) to 50 μg of protein and loaded onto 12% polyacrylamide gels (Bio-Rad, 4561041). Proteins were separated by SDS-PAGE (100 V) using an SDS running buffer (Bio-Rad, 1610772) and then transferred onto a nitrocellulose membrane using a Tris-Glycine Transfer Buffer (Bio-Rad, 1610771) (250 mA) for 1 h. Membranes were blocked 1 h at RT with 5% fat-free milk or 3% bovine serum albumin (BSA) in 1× TBS-T (0.14 M NaCl, 0.02 M Tris base, pH 7.6, supplemented with 0.05% Tween20) and incubated overnight at 4°C with primary antibodies. The following primary antibodies were used: mouse monoclonal anti-DDK-myc (FLAG) (1:1000; Origene, TA180144), rabbit monoclonal anti-pan-VDAC (1:1000; Abcam, ab154856), rabbit monoclonal anti-GPX4 (1:1000; Abcam, ab125066), rabbit polyclonal anti-Calreticulin (1:20 000; Sigma-Aldrich, C4606), rabbit polyclonal anti-4-hydroxynonenal (1:1000; Abcam, ab46545), mouse monoclonal anti-Hsp70 (1:1000; BD Transduction Laboratories, H53220). Then, membranes were washed three times for 10 min in 1× TBS-T and incubated 1 h at RT with horseradish peroxidase conjugated secondary antibodies. The following secondary antibodies were used: HRP-conjugated goat anti-Mouse IgG (1:5000; Jackson ImmunoResearch, 115-035-146); HRP-conjugated goat anti-Rabbit IgG (1:5000; Jackson ImmunoResearch, 111-035-144). The immunoreactive bands were visualized with Clarity Western ECL Substrate (Bio-Rad, 1705061) using a Kodak Gel Logic imaging system (Kodak). When performed, band signal intensities were quantified by densitometry using ImageJ [25].

### 2.8. Iron quantification assay

Iron concentration was measured using an Iron Assay Kit (Sigma-Aldrich, MAK025) following manufacturer instructions. Briefly, cells (2 × 10^6^) were homogenized in 4–10 volumes of assay buffer and centrifuged at 16 000 × *g* for 10 min at 4°C to remove insoluble material. Samples were resuspended in a final volume of 100 µl. To measure ferrous iron (Fe^2+^) and total iron, 5 µl of assay buffer or 5 µl of iron reducer was added to 30 µl of samples in a 96-well plate, respectively. In parallel, the iron standard curve was prepared following the manufacturer procedure. Then, samples were diluted up to 100 µl of total volume with assay buffer and incubated under constant shaking for 30 min at 25°C, protected from light. Further, 100 µl of iron probe was added to each well containing standards and samples. Reactions were incubated under constant shaking for 60 min at 25°C. The absorbance at 593 nm was measured using a VICTOR^3^ 1420 Multilabel Counter plate reader (Perkin-Elmer). Fe^2+^ and total iron (Fe^2+^+ Fe^3+^) concentrations were obtained from a standard curve. Fe^3+^ concentration was derived from the subtraction of Fe^2+^ concentration from total iron. Iron fractions were obtained considering the concentrations of Fe^2+^ and Fe^3+^ as percentages of total iron.

### 2.9. Spheroid generation

To set up 3D cultures, cells (SKOV3cis^mock^ and SKOV3cis^DNAJC15-OE^: 4000 cells per well; OC314^scramble^ and OC314^DNAJC15-KD^: 5000 cells per well) were seeded into ultra-low attachment 96-well round bottom plates (Corning, 7007) in 200 μl per well. After seeding, SKOV3cis cells spontaneously aggregate to form 3D structures, while for OC314 a centrifugation step (500 × *g* for 5 min) was performed. Plates were placed at 37°C with 5% CO_2_ for 4 days to allow spheroid formation. Starting from the fourth day after seeding (time 0, T0), spheroids’ growth was monitored for 10 days. Spheroids at 0, 5 and 10 days were visualized with a digital imaging system using an inverted microscope with a 10× objective (0.25 numerical aperture, NA) (Nikon Eclipse Ti-U, Nikon). Images were captured using a Basler Ace2 camera equipped with a Sony IMX546 CMOS sensor. Major (*a*) and minor (*b*) axes were measured for each spheroid with ImageJ and volumes were calculated approximating spheroid shape to an ellipsoid with depth = width (*V* = 4/3π*ab*^2^).

### 2.10. Lipid droplet staining

Cells (15 × 10^4^ per dish) were seeded on glass cover slides (Ø 10 mm) and incubated with 2 ml of culture medium. After 24 h, cells were treated with 2 µM Nile Red (Sigma-Aldrich, N3013) for 30 min at 37°C. Nuclei were stained with 1 µg ml^−1^ Hoechst 33342 (Molecular Probes, H1399) incubating cells for 30 min at 37°C. After the incubation, cells were washed with PBS, and the slide was placed in a specific metal grid with 1 ml of DMEM without red phenol supplemented with 25 mM HEPES (Gibco™ Thermo Fisher Scientific, 21063029). Lipid droplets (LDs) were visualized with a digital imaging system using an inverted epifluorescence microscope with a 60× (1.4 NA) oil objective (Nikon Eclipse Ti-U, Nikon). Images were captured with a Photometrics BSI PRIME Express camera system (Teledyne Photometrics) and elaborated with NIS-Elements Software (Nikon). The number of LDs was quantified by using the MRI_Lipid_droplets_tool (https://dev.mri.cnrs.fr/projects/imagej-macros/wiki/Lipid_Droplets_Tool) and normalized to the number of cells per image.

### 2.11. Glutathione peroxidase 4 activity determination

Cells (4 × 10^6^) were resuspended (10 × 10^6^ cells ml^−1^) in lysis buffer (0.1 M KPi (pH 7.4), 0.15 M KCl, 0.05% CHAPS), supplemented with 5 mM β-mercaptoethanol and protease inhibitors, and rapidly homogenized using a glass/Teflon Potter-Elvehjem homogenizer. Cell extracts were centrifugated at 15 000 × *g* at 4°C for 20 min, supernatants were collected, and an aliquot was used to measure the protein concentration with the Bradford method. The enzyme assay was carried out in quartz cuvettes with magnetic stirring, by using a spectrophotometer (V550 Jasco). Cuvettes were prepared reaching 1 ml of final volume with assay buffer (0.1 M KPi (pH 7.8), 5 mM EDTA, 0.1% (v/v) Triton X-100) supplemented with 5 mM GSH (Sigma-Aldrich, G6529), 160 µM NADPH (Sigma-Aldrich N1630), 25.6 U ml^−1^ glutathione reductase (Sigma-Aldrich, G3664), 200 µM *tert*-butyl hydroperoxide (Sigma-Aldrich, 458139) and incubating the reaction for 5 min at room temperature. Then, time course kinetics of the non-specific reaction was detected. Sample supernatant (100 µl) was added to start the glutathione peroxidase 4 (GPX4) enzyme-specific reaction and NAPDH absorbance was measured at 340 nm (*ε*_340_ = 6.22 mM^−1^ cm^−1^). Specific enzyme activities were normalized on protein amount and expressed as µM min^−1^ mg^−1^ protein.

### 2.12. GSSG/GSH determination

The intracellular GSSG and GSH concentrations were measured by an enzymatic assay as previously described in [26]. Briefly, cells (8 × 10^6^) were resuspended in 900 µl of 6% metaphosphoric acid (Riedel-de Haën, 04103) and incubated in ice for 5 min. Then, samples were centrifuged at 10 000 × *g* for 5 min and supernatants were collected for the subsequent assays. For total GSH determination, 100 µl of samples diluted 1:20 in 6% metaphosphoric acid was added to 860 µl of 0.1 M KH_2_PO_4_ (pH 7.4) with 5 mM EDTA buffer supplemented with 0.5 mM DTNB (Sigma-Aldrich, D8130) and 0.4 mM NADPH (Sigma-Aldrich, N1630), placed in a 24-well plate and reaction was started by adding 2 U of glutathione reductase (Sigma-Aldrich, G3664). The reaction was recorded continuously at 405 nm at 37°C with a VICTOR^3^ 1420 Multilabel Counter plate reader (Perkin-Elmer). The total amount of GSH in the samples was determined by using a GSH (Sigma-Aldrich, G6529) standard curve in the range of 1–4 µg ml^−1^. For GSSG determination, 200 µl of sample was treated for derivatization with 8 µl of 2-vinylpyridine (Sigma-Aldrich, 132292) and 12 µl of triethanolamine (Sigma-Aldrich, T58300). The mixtures were vigorously mixed and incubated at room temperature for 60 min. Then, 100 µl of sample was assayed as previously described for total GSH determination. The amount of GSSG in the samples was determined by using a GSSG (Sigma-Aldrich, G6654) standard curve in the range of 0.25–1 µg ml^−1^. Total glutathione and GSSG data (nmol mg^−1^) were normalized to sample protein amount (mg ml^−1^). Finally, GSH was calculated by subtracting GSSG from total GSH and data were recorded as GSSG/GSH ratio.

### 2.13. Statistical analysis

Statistical analyses were performed using GraphPad Prism v.8 (GraphPad Software Inc., San Diego, CA, USA). Data were expressed as mean ± SEM as specified in each panel. Unless stated otherwise, two-tailed unpaired or paired Student’s *t*-test assuming equal variances was performed and at least three biological replicates were conducted for each experiment. Statistical significance was defined by *p* ≤ 0.05.

## 3. Results

### 3.1. High DNAJC15 levels sensitize ovarian cancer cells to cisplatin and decrease their proliferative capability

**Figure 1.**
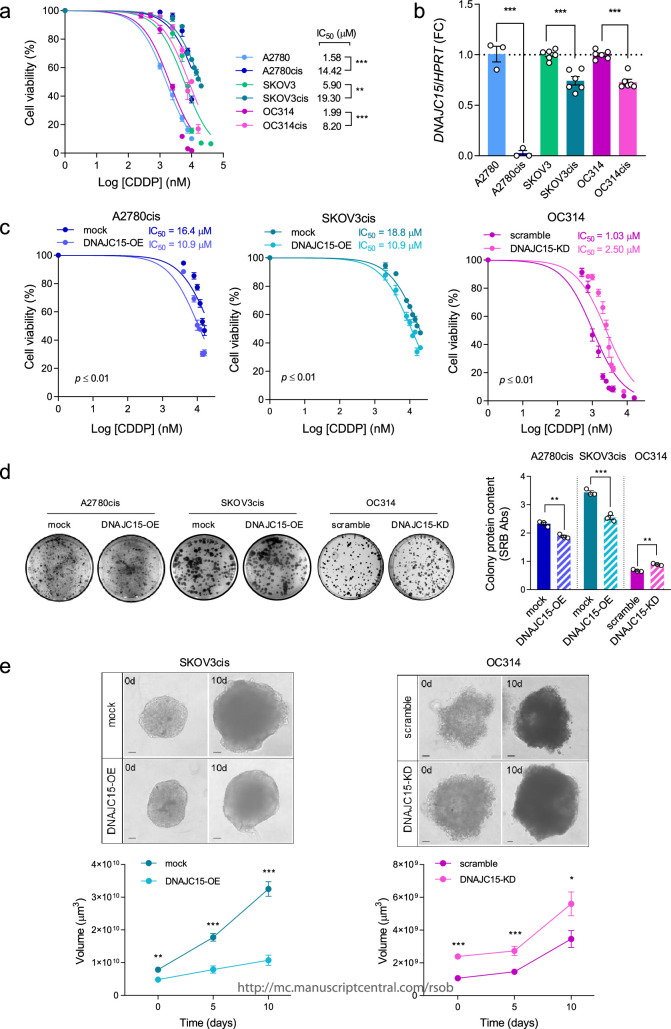
High DNAJC15 levels sensitize OC cells to CDDP and decrease their proliferative capability. (*a*) IC_50_ was determined after 72 h of CDDP treatment by measuring cell viability using SRB assay. Data (mean ± SEM; *n *≥ 3) are expressed as % considering untreated cells as 100%. (*b*) *DNAJC15* mRNA expression levels were evaluated using qRT-PCR analysis. Hypoxanthine phosphoribosyl transferase 1 (*HRPT*) was used as housekeeping gene. Data (mean ± SEM; *n *≥ 3) are shown as fold change (FC). (*c*) IC_50_ was determined after 72 h of CDDP treatments in DNAJC15-OE and -KD OC cell lines and their respective controls (mock and scramble) by measuring cell viability using SRB assay. Data (mean ± SEM; *n* = 3) are expressed as % considering untreated cells as 100%. (*d*) Colony formation assay of DNAJC15-OE and -KD OC cell lines. Images of a representative experiment are shown. Colony protein content was obtained using SRB assay and data are reported as mean ± SEM (*n* = 3). (*e*) 3D cultures derived from SKOV3cis (DNAJC15-OE and mock) and OC314 (DNAJC15-KD and scramble) cell lines. 3D cultures were monitored for 10 days (10d) after spheroid formation (4 days from the seeding, 0d). Images of a representative experiment are shown (scale bar, 100 μm) and volumes of spheroids are reported as mean ± SEM (*n *> 3). Statistical significances were calculated with Student’s *t*-test (*p ≤ 0.05; **p ≤ 0.01; ***p ≤ 0.001).

To evaluate whether DNAJC15 contributes to platin derivative resistance in OC, we exploited a panel of syngeneic CDDP resistant (cis) and sensitive cells, namely A2780, SKOV3 and OC314. The half-maximal inhibitory concentration (IC_50_) for CDDP was significantly higher in the resistant cells compared with their sensitive counterparts (figure 1*a*). Interestingly, a significant decrease of *DNAJC15* mRNA levels was observed in CDDP-resistant compared with sensitive cells (figure 1*b*), suggesting that DNAJC15 downregulation may be positively correlated with the acquisition of CDDP resistance. To prove a causal link between DNAJC15 and CDDP sensitivity, we generated OC cell lines in which DNAJC15 was either overexpressed or knocked down. First, we transduced the most CDDP-resistant A2780cis and SKOV3cis cells with empty- or DNAJC15-DDK-myc tagged vectors (hereafter referred to as mock and DNAJC15-OE cells, respectively). Overexpression of DNAJC15 resulted in the increase of both mRNA (electronic supplementary material, figure S1*a*) and protein levels in mitochondrial fractions (electronic supplementary material, figure S1*b*), indicating the correct localization of the exogenous chaperone. DNAJC15 was instead downregulated in the most CDDP-sensitive OC314 cell line by using shRNAs. Among the different shRNAs tested, the most efficient in decreasing *DNAJC15* levels was shDNAJC15d (electronic supplementary material, figure S1*c*), which was hence used in all subsequent experiments (hereafter referred to as DNAJC15-KD). Notably, both DNAJC15-OE cell lines showed a significantly reduced CDDP IC_50_ compared with mock, while DNAJC15-KD cells increased their resistance (figure 1*c*), suggesting that DNAJC15 modulates sensitivity to CDDP in OC cells. Since resistance to antiblastic therapy is a function of the proliferative capability of cancer cells, we investigated whether DNAJC15 expression may affect colony and spheroid formation. Clonogenic capacity was decreased in both DNAJC15-OE cell lines compared with mock, while DNAJC15-KD cells showed an increased ability to form colonies compared with their empty vector control (scramble) (figure 1*d*). These observations were mirrored in 3D cultures since spheroids derived from SKOV3cis^DNAJC15-OE^ cells displayed a significant reduction of their volume, whereas the latter was increased in those obtained from OC314^DNAJC15-KD^ (figure 1*e*). Notably, DNAJC15 levels impacted both spheroids’ formation (0 days) and growth (5 and 10 days) (figure 1*e*). Overall, these data suggest that high DNAJC15 expression levels increase sensitivity to CDDP in association with lower *in vitro* proliferative capabilities of OC cells.

### 3.2. High levels of DNAJC15 trigger ferroptosis in cisplatin-resistant ovarian cancer cells

**Figure 2.**
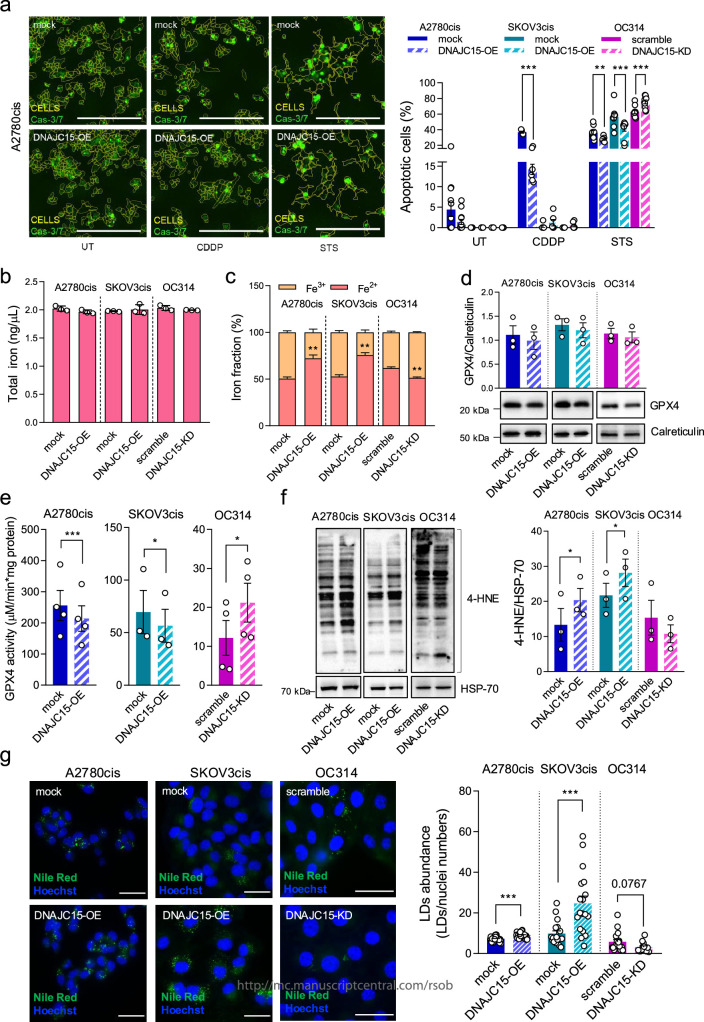
High levels of DNAJC15 trigger ferroptosis in CDDP-resistant OC cells. (*a*) Representative images (insets of larger pictures; scale bars, 400 µm) and quantification of apoptotic cells after 72 h of growth in absence of treatments (UT), upon CDDP (8 µM) and staurosporine (STS, 100 nM) treatments. Data (mean ± SEM; *n *≥ 3) are expressed as percentage and were obtained dividing the number of positive green cells (Cas-3/7) by the total number of cells per field. Statistical significance was calculated with Student’s *t*-test (*p ≤ 0.05; **p ≤ 0.01; ***p ≤ 0.001). (*b*) Total iron levels expressed as ng µl^−1^. Data are mean ± SEM (*n* = 3). (*c*) Iron (Fe^2+^ and Fe^3+^) fractions expressed in percentage. Data are mean ± SEM (*n* = 3). Statistical significance was calculated with Student’s *t*-test (**p ≤ 0.01). (*d*) Western blot analysis of GPX4 levels in total protein extract. Calreticulin was used as loading control. A representative experiment is shown and densitometric data are reported as mean ± SEM (*n* = 3). (*e*) GPX4 enzymatic activity. Data (mean ± SEM; *n *≥ 3) were normalized to total protein amount (mg). Statistical significance was calculated with paired Student’s *t*-test (**p ≤ 0.01; ***p ≤ 0.001). (*f*) Western blot analysis of 4-HNE protein-adducts. A representative experiment is shown and densitometric data are expressed as mean ± SEM (*n* = 3). HSP-70 was used as loading control. Statistical significance was calculated with paired Student’s *t*-test (*p ≤ 0.05). (*g*) Representative images of lipid droplets (LDs) and nuclei in DNAJC15-OE and -KD cell lines compared with mock and scramble counterparts, obtained by using Nile Red (LDs, green) and Hoechst (nuclei, blue) staining, respectively. Scale bar, 50 μm. LD abundance (mean ± SEM) was expressed as ratio between LDs and nuclei numbers per field (at least 10 different fields for *n *≥ 3 biological replicates). Statistical significance was calculated with Student’s *t*-test (***p ≤ 0.001).

It has been previously reported that DNAJC15 overexpression can increase the apoptotic cell death sensitivity in different cancer models upon CDDP treatment possibly due to the opening of MPTP [5]. Hence, we explored this mechanism using DNAJC15-high and -low OC cells in absence or presence of CDDP for 72 h (figure 2*a*; electronic supplementary material, figure S1*d*,*e*). The analysis of caspase 3/7 activation showed that high levels of DNAJC15 (A2780cis^DNAJC15-OE^, SKOV3cis^DNAJC15-OE^ and OC314^scramble^) did not induce apoptosis *per se* (figure 2*a*; untreated cells, UT). Albeit CDDP induced apoptotic events in the A2780cis lines, DNAJC15-high cells were not more prone to apoptosis activation compared with DNAJC15-low counterparts (A2780cis^mock^) (figure 2*a*; CDDP), indicating that enhanced sensitivity to CDDP induced by DNAJC15 is not related to its ability to trigger apoptosis. Notably, high DNAJC15 (DNAJC15-OE or scramble cells) levels made OC cells less sensitive to staurosporine (STS), a well-known apoptosis activator, suggesting that DNAJC15 safeguards against this type of cell death (figure 2*a*; STS). Since these data revealed that high DNAJC15 levels did not trigger apoptosis in response to CDDP treatment, we were prompted to look for alternative cell death mechanisms modulating DNAJC15-mediated CDDP sensitivity. In this frame, it has been demonstrated that ferroptosis activation potentiates the anti-tumour effect of conventional chemotherapy and radiotherapy [22,27]. The role of DNAJC15 in regulating ferroptosis in OC cells upon CDDP treatment has not been dissected so far. Among the canonical ferroptosis markers, we first evaluated intracellular iron levels and its redox state (figure 2*b*,*c*). While modulation of DNAJC15 did not affect the total iron levels (figure 2*b*), an unbalance towards the Fe^2+^ form was observed in DNAJC15-OE cells (figure 2*c*). Accordingly, downregulation of DNAJC15 induced a decrease in Fe^2+^ iron (figure 2*c*), thus setting the basis for the hypothesis of a DNAJC15-mediated activation of ferroptosis. We next evaluated the expression levels and the activity of GPX4, a well-known enzyme of ferroptosis attenuation [28]. Although no significant change was observed in protein levels (figure 2*d*), its catalytic activity was reduced in DNAJC15-OE cells compared with mock counterparts (figure 2*e*). Coherently, GPX4 activity was increased in DNAJC15-KD cells compared with scramble (figure 2*e*), suggesting that high levels of DNAJC15 may promote ferroptosis likely by blocking the protective activity of this enzyme. These data were corroborated by the decrease in GSSG/GSH ratio particularly in DNAJC15-OE cells (electronic supplementary material, figure S1*f*). However, no change in GSSG/GSH ratio was observed in DNAJC15-KD cells likely due to the presence of the residual *DNAJC15* levels (electronic supplementary material, figure S1*c*). Since he GSH–GPX4 axis is mainly implicated in maintaining the reduced status of lipids, we evaluated the abundance of 4-hydroxynonenal (4-HNE), the end-product of lipid peroxidation and indicator of an overall oxidative stress [29,30]. The levels of 4-HNE were significantly increased in DNAJC15-OE cells compared with their mock counterparts, and in agreement with GSSG/GSH ratio, no change in 4-HNE amount was observed in DNAJC15-KD cells (figure 2*f*). Another mechanism to buffer the excess of damaged lipids is to confine them into lipid droplets (LDs) [31,32]. In this frame, a significant increase of these organelles was observed in DNAJC15-OE cells compared with mock and a trend towards their reduction was found in DNAJC15-KD cells compared with scramble (figure 2*g*), suggesting that DNAJC15-related decrease in GPX4 activity may foster LD biogenesis in the attempt to reduce membrane peroxidation. Taken together, these data corroborate our hypothesis that high DNAJC15 levels may be able to intrinsically trigger ferroptosis sensitizing OC cells to CDDP.

### 3.3. DNAJC15-dependent lipid peroxidation triggers ferroptotic isplatin sensitivity

**Figure 3.**
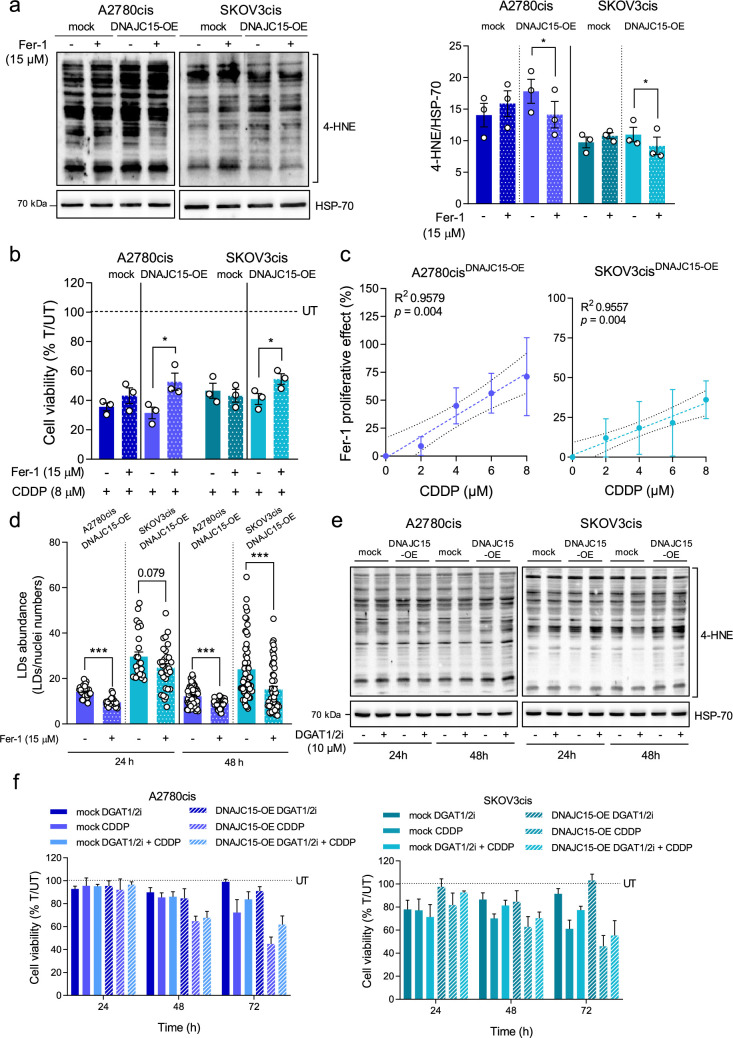
DNAJC15-dependent lipid peroxidation triggers ferroptotic CDDP sensitivity. (*a*) Western blot analysis of 4-HNE protein-adducts with and without ferrostatin-1 (Fer-1) treatment (15 µM for 24 h). A representative experiment is shown and densitometric data are expressed as mean ± SEM (*n* = 3). HSP-70 was used as loading control. Statistical significance was calculated with paired Student’s *t*-test (*p ≤ 0.05; **p ≤ 0.01). (*b*) Cell viability of DNAJC15-OE and mock cells upon CDDP treatment (8 µM) with (T) and without (UT) Fer-1 (15 µM) for 24 h. Data (mean ± SEM; *n* = 3) are expressed as % considering UT as 100%. Statistical significance was calculated with Student’s *t*-test (*p ≤ 0.05). (*c*) Correlation between Fer-1 treatment effect and CDDP concentrations in DNAJC15-OE cells. Data (mean ± SEM; *n* = 3) were expressed as cell viability (%) upon the combined treatment with Fer-1 (15 µM) and with different CDDP concentrations. CDDP untreated condition was considered as 100%. The Fer-1 proliferative effect was calculated subtracting the % of viable cells upon the combined treatment from that of the Fer-1 untreated condition. Statistical significance, calculated with Student’s *t*-test, and *R*^2^ values are reported for each graph. (*d*) Lipid droplet (LD) abundance in DNAJC15-OE cells after 24 and 48 h of Fer-1 (15 µM) treatment. Data (mean ± SEM) are expressed as ratio between LDs and nuclei numbers per field (at least 10 different fields for *n *≥ 3 biological replicates). Statistical significance was calculated with Student’s *t*-test (***p ≤ 0.001). (*e*) Western blot analysis of 4-HNE protein-adducts with and without DGAT1/2 inhibitor (DGAT1/2i) treatments (10 µM for 24 h and 48 h). HSP-70 was used as loading control. Representative experiment of three is shown. (*f*) Cell viability of DNAJC15-OE cells upon treatments with DGAT1/2i alone (10 µM), CDDP alone (8 µM) or their combination. Data (mean ± SEM; *n* = 3) are expressed as % considering untreated cells (UT) as 100%.

To dissect the relationship among DNAJC15, peroxidized lipids, LD biogenesis and ferroptotic CDDP sensitivity, we exploited Ferrostatin-1 (Fer-1), a widely used lipid peroxidation inhibitor [33], and assessed its effects on cell viability after treatment with CDDP. Fer-1 treatment induced a significant decrease of lipid peroxidation in cells overexpressing DNAJC15, as indicated by the 4-HNE levels (figure 3*a*) but did not impact growth ability in 2D OC cells and 3D cultures (electronic supplementary material, figure S2*a*,*c*). Conversely, when cells were treated with CDDP, cell viability increased upon Fer-1 treatment specifically in DNAJC15-OE cells (figure 3*b*). Notably, the differences in terms of viability between Fer-1 treated and untreated cells in the presence of CDDP positively correlated with increasing CDDP concentration in DNAJC15-OE cells (figure 3*c*). These data were mirrored in 3D cultures since spheroids derived from SKOV3cis^DNAJC15-OE^ cells displayed an increase in the ability of spheroid formation and growth upon treatment with CDDP plus Fer-1 when compared with CDDP treatment alone (electronic supplementary material, figure S2*d*,*e*). These findings suggest that ferroptosis activation driven by DNAJC15-dependent lipid peroxidation has a priming effect for raising OC cell vulnerability to CDDP. Then, to understand whether DNAJC15-dependent LD biogenesis was related to lipid peroxidation, LD abundance was measured in DNAJC15-OE cells treated or not with Fer-1. The abundance of these organelles was indeed significantly reduced upon Fer-1 treatment in both DNAJC15-OE cell lines (figure 3*d*; electronic supplementary material, figure S2*b*), pointing to a direct link between DNAJC15-driven lipid peroxidation and LD formation. Since it has been widely reported that the accumulation of LDs is a crucial salvage mechanism to alleviate cellular lipotoxic stress [34], we determined CDDP sensitivity preventing LD formation in OC cells. Incubation of both mock and DNAJC15-OE cells with a combination of specific inhibitors of diacylglycerol acyltransferase 1/2 (DGAT1/2) [35–37] led to markedly reduced LD amount (electronic supplementary material, figure S3*a*,*b*) but did not influence the levels of 4-HNE even after prolonged treatment (figure 3*e*; electronic supplementary material, figure S3*c*). Moreover, DGAT1/2 inhibitors alone or in combination with CDDP did not affect cell viability regardless of the DNAJC15 expression levels (figure 3*f*), allowing to exclude the accumulation of LDs induced by DNAJC15 overexpression as a possible modulator of CDDP sensitivity, at least in OC cells.

## 4. Discussion

In this study, we showed how high DNAJC15 expression enhances OC cells’ vulnerability to CDDP toxicity by promoting ferroptosis activation. Starting from the observations regarding the involvement of DNAJC15 in the acute response to CDDP [5] and its identification as a prognostic factor in OC patients [14], we observed that DNAJC15 expression levels positively correlated with CDDP acute sensitivity and inversely with acquired drug resistance in OC cells. Indeed, while DNAJC15 overexpression increased CDDP susceptibility and dampened proliferative properties of chemoresistant OC cells, its downregulation counteracted these effects in chemosensitive cells. Hence, the dissection of DNAJC15-linked molecular mechanisms behind sensitivity to platinum-based chemotherapy may unveil novel insights useful to overcome the main clinical hurdles of OC in terms of therapeutic failure and chemoresistance onset [38]. Taking advantage from OC cells in which DNAJC15 was overexpressed, we noted that the levels of this chaperonin directly correlated with increased amount of lipid peroxidation and decreased antioxidant defences. Particularly, we showed a significant drop in the activity of GPX4, a peroxidase that uses glutathione as donor substrate to reduce lipid hydroperoxides producing lipid alcohols. Such enzyme is crucial in regulating cellular oxidative state since its low levels and/or activity result in lipid peroxidation boosting with a subsequent promotion of membrane damage and ferroptosis [39]. In line with our data, it has been reported that ferroptosis activation enhances the susceptibility to standard platinum and taxane-based chemotherapy in OC, thus exerting an anti-tumour effect [40–42]. Moreover, the DNAJC15-linked accumulation of Fe^2+^, the main indicator of ferroptosis, supports ferroptosis-driven CDDP sensitivity in OC cells. Interestingly, DNAJC15 overexpression has been reported to induce oxidative stress by modulating the mitochondrial respiratory chain function [5]. This finding, together with GPX4 reduced activity and iron accumulation, may explain the boost in lipid peroxidation with subsequent ferroptosis activation that we found in the presence of high DNAJC15 levels in OC cells. Indeed, the observation that Fer-1 treatment reduced both lipid peroxidation and CDDP toxicity supports our hypothesis that DNAJC15 is potentially responsible for the activation of ferroptosis, which likely increased the sensitivity of OC cells to CDDP. Recently, it has been reported that metastatic cancer cells display a higher susceptibility to ferroptosis-mediated cell death and that mitochondria play a central role in ferroptosis by regulating various processes such as iron metabolism, reactive oxygen species production, energy metabolism involving respiratory chain activity and tricarboxylic cycle acid metabolites and enzymes [22,23]. DNAJC15 has been described as a mitochondrial chaperonin involved in the formation of a specific TIMM23 translocation complex, which in stressing conditions specifically transports proteins that lack canonical mitochondrial pre-sequences [5]. Therefore, we suggest that overexpression of DNAJC15 may promote the transport of proteins relevant in the mitochondrial regulation of ferroptosis [43], making cells more prone to this type of cell death. Notably, our results differ from those of Sinha *et al.* [5], in which DNAJC15 overexpression in cancer cells resulted in apoptosis induction mediated by the MPTP opening. However, these observations highlight a context-dependent role of DNAJC15 in activating different death pathways that may account for its involvement in the regulation of cell viability and proliferation crucial for cancer therapy sensitivity and response.

The finding that DNAJC15-overexpressing cells showed a marked increase in LDs deserves further investigation. Although we have excluded their involvement in the modulation of CDDP sensitivity, their presence is clearly correlated with high DNAJC15 expression and lipid peroxidation increase. One possible explanation is that DNAJC15 plays a role in fatty acid oxidation. Indeed, it has been reported that DNAJC15 knock-out mice showed increased resistance to hepatic steatosis induced by a high-fat diet as model for non-alcoholic fatty liver disease [4]. This resistance was correlated with increased fatty acid oxidation and oxidative phosphorylation in liver. Conversely, overexpression of DNAJC15 may favour the accumulation of peroxidized lipids triggering a cellular lipotoxic stress that may be alleviated by the observed LD increase in OC cell lines. However, LD biogenesis prevention did not impact CDDP response indicating that their accumulation is not sufficient to allow OC cells to overcome ferroptosis.

**Figure 4.**
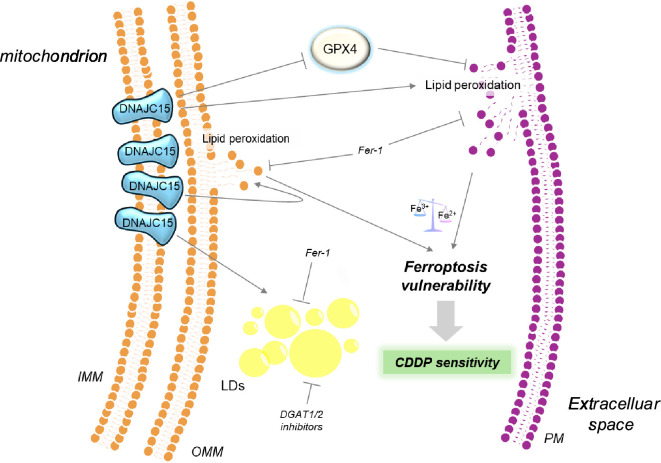
High levels of DNAJC15 promote vulnerability to ferroptosis of chemoresistant OC cells. OC cells with high DNAJC15 levels display lower ferroptosis antioxidant defence (GPX4) and an unbalance towards the Fe^2+^ form leading to lipid membrane damage (lipid peroxidation) that in turn favours the accumulation of lipid droplets (LDs) to alleviate cellular lipotoxic stress. In this scenario, the boost of lipid peroxides overcomes the defence system capability triggering ferroptosis activation. This unprogrammed cell death enhances OC cells’ vulnerability to CDDP toxicity pointing out DNAJC15 as a mitochondrial protein with an antiblastic effect in OC.

In conclusion, for the first time, we reported that DNAJC15 can trigger ferroptosis in OC cell lines by augmenting lipid peroxidation and lowering antioxidant defences (figure 4). This led us to envision this chaperonin as a mitochondrial protein that has an antiblastic effect in OC, caused by increased susceptibility to ferroptosis, especially since its expression inversely correlates with CDDP resistance and proliferation. These observations underline the role of DNAJC15 as a prognostic factor in OC. Particularly, the unveiling of a DNAJC15-linked ferroptosis activation mechanism could be useful for the identification of additional molecular determinants involved in this cell death pathway, which has recently been shown to contribute to tumour suppression. Therefore, DNAJC15-mediated induction of ferroptosis may provide new opportunities for the development of molecules that could reverse chemotherapy resistance in OC.

## Data Availability

The data are accessible on a public repository [44]. Supplementary material is available online [45].
